# Comparative Proteomic Analysis of *Desulfotomaculum reducens* MI-1: Insights into the Metabolic Versatility of a Gram-Positive Sulfate- and Metal-Reducing Bacterium

**DOI:** 10.3389/fmicb.2016.00191

**Published:** 2016-02-19

**Authors:** Anne E. Otwell, Stephen J. Callister, Erika M. Zink, Richard D. Smith, Ruth E. Richardson

**Affiliations:** ^1^Department of Microbiology, Cornell UniversityIthaca, NY, USA; ^2^Pacific Northwest National Laboratory, Biological Sciences DivisionRichland, WA, USA; ^3^Department of Civil and Environmental Engineering, Cornell UniversityIthaca, NY, USA

**Keywords:** Fe(III) reduction, sulfate reduction, *Desulfotomaculum reducens*, gram-positive bacteria, comparative proteomic analysis, flavin-based electron bifurcation, heterodisulfide reductase

## Abstract

The proteomes of the metabolically versatile and poorly characterized Gram-positive bacterium *Desulfotomaculum reducens* MI-1 were compared across four cultivation conditions including sulfate reduction, soluble Fe(III) reduction, insoluble Fe(III) reduction, and pyruvate fermentation. Collectively across conditions, we observed at high confidence ~38% of genome-encoded proteins. Here, we focus on proteins that display significant differential abundance on conditions tested. To the best of our knowledge, this is the first full-proteome study focused on a Gram-positive organism cultivated either on sulfate or metal-reducing conditions. Several proteins with uncharacterized function encoded within heterodisulfide reductase (*hdr*)-containing loci were upregulated on either sulfate (Dred_0633-4, Dred_0689-90, and Dred_1325-30) or Fe(III)-citrate-reducing conditions (Dred_0432-3 and Dred_1778-84). Two of these *hdr*-containing loci display homology to recently described flavin-based electron bifurcation (FBEB) pathways (Dred_1325-30 and Dred_1778-84). Additionally, we propose that a cluster of proteins, which is homologous to a described FBEB lactate dehydrogenase (LDH) complex, is performing lactate oxidation in *D. reducens* (Dred_0367-9). Analysis of the putative sulfate reduction machinery in *D. reducens* revealed that most of these proteins are constitutively expressed across cultivation conditions tested. In addition, peptides from the single multiheme c-type cytochrome (MHC) in the genome were exclusively observed on the insoluble Fe(III) condition, suggesting that this MHC may play a role in reduction of insoluble metals.

## Introduction

Microbial sulfate and metal reduction are thought to be ancient processes that today are major drivers of nutrient cycles in anaerobic environments (Bird et al., 2011; Pereira et al., 2011; Hori et al., 2015). Decades of research have focused on understanding the proteins involved in these electron transfer pathways, and Gram-negative bacteria have mainly served as model organisms in studies. However, it is becoming increasingly understood that sulfate-reducing organisms (SROs) and dissimilatory metal-reducing organisms (DMROs) are phylogenetically diverse, and Gram-positive bacteria are suspected to be major contributors to these processes in natural settings (Petrie et al., 2003; Suzuki et al., 2003; Cardenas et al., 2010; Williamson et al., 2013; Newsome et al., 2014).

Studies on the Gram-negative genus *Desulfovibrio* have been foundational in elucidating mechanisms involved in microbial sulfate reduction. Core proteins in the sulfate reduction pathway (sulfate adenyltransferase, APS reductase, sulfite reductase) are conserved amongst SROs. However, several other proteins described in the pathway of sulfate reduction in *Desulfovibrio* do not contain homologs in the genomes of Gram-positive SROs, implying that divergent mechanisms are involved (Pereira et al., 2011; Grein et al., 2013). Even less is understood regarding metal reduction in Gram-positive bacteria. Studies on Gram-negative metal-reducing representatives, namely *Geobacter* and *Shewanella* species, have defined the current understanding of microbial metal reduction. A critical trait for metal reduction shared amongst model metal reducers is an abundance of genes encoding multiheme c-type cytochromes (MHCs; Wall and Krumholz, 2006; Shi et al., 2009; Sharma et al., 2010). Gram-positive bacteria, however, rarely contain multiple MHCs (Sharma et al., 2010).

The *Desulfotomaculum* genus is composed of anaerobic, Gram-positive, endospore-forming SROs. These Peptococcaceae are widely distributed in the environment, and the number of identified species as well as sequenced genomes is continually expanding (Aüllo et al., 2013; Kuever et al., 2014; Visser et al., 2014). Characterized species display considerable metabolic versatility, and some have been shown to reduce metals (Tebo and Obraztsova, 1998; Haouari et al., 2008; Barton et al., 2015). Certain *Desulfotomaculum* species completely oxidize organic substrates to CO_2_, whereas others are incomplete oxidizers. The sulfite reductases from these two groups are phylogenetically distinct, and it has been suggested that complete oxidizers acquired their sulfite reductase laterally from a deltaproteobacterial donor (Klein et al., 2001; Zverlov et al., 2005).

*Desulfotomaculum reducens* MI-1 was isolated from a heavy-metal contaminated site and is a species of marked interest as a Gram-positive SRO that also has the capability to reduce metals including Fe(III) and Mn(IV; Tebo and Obraztsova, 1998). As one of only a few organisms reported to conserve energy from utilization of U(VI) and Cr(VI) as electron acceptors, *D. reducens* is also of interest for bioremediation applications (Tebo and Obraztsova, 1998; Wall and Krumholz, 2006; Barton et al., 2015). Previous work in *D. reducens* has helped to elucidate the mechanism of Fe(III) reduction. It was proposed that Fe(III)-oxide reduction during pyruvate fermentation occurs through a soluble electron shuttle, as direct contact was found to not be required for Fe(III) reduction on this condition (Dalla Vecchia et al., 2014b). Conversely, direct contact was found to be required for Fe(III)-oxide reduction with lactate as electron donor and is therefore not mediated by a soluble shuttle (Dalla Vecchia et al., 2014a). Understanding of the proteins involved in Fe(III) reduction in *D. reducens* is less developed. The genome contains one annotated MHC (Dred_0700-1, nitrite reductase), a likely candidate for Fe(III) reduction (Junier et al., 2010). However, studies have analyzed its expression levels and found it to not be induced in the presence of soluble Fe(III) or U(VI) relative to pyruvate fermentation conditions (Junier et al., 2011; Dalla Vecchia et al., 2014a,b). One of these studies also analyzed the surface proteins (or surfaceome) of *D. reducens* while fermenting pyruvate or reducing soluble Fe(III) with lactate as electron donor and identified no peptides for the MHC (Dalla Vecchia et al., 2014a). However, no mRNA/protein-based analyses of the MHC during insoluble Fe(III) reduction have been performed.

In this study, we compared the proteomes of *D. reducens* during sulfate, soluble Fe(III), and insoluble Fe(III) reduction (all with lactate as electron donor), and pyruvate fermentation. The genome of *D. reducens* was sequenced in 2010, allowing for genomic predictions to be tested with proteomic measurements (Junier et al., 2010). The *D. reducens* genome contains an abundance of proteins that contain oxidoreductase-related annotations but lack specific functional annotations. There is increasing understanding of the types of domains and proteins that drive anaerobic respiration (Grein et al., 2013). In *D. reducens*, however, experimental information is not available in order to determine the function of many predicted redox proteins. Furthermore, in recent years, pathways of flavin-based electron bifurcation (FBEB) have been described in anaerobic microorganisms. FBEB is now regarded as a third mode of energy conservation, along with respiration and fermentation (Herrmann et al., 2008; Buckel and Thauer, 2013). The *D. reducens* genome contains homology to described FBEB pathways, and comparative proteomic analysis provides a tool for predicting whether these pathways are contributing to the metabolism of *D. reducens*. We report here the first global proteomic comparison of a *Desulfotomaculum* species. In fact, to the best of our knowledge this is the first full-proteome comparative analysis of any Gram-positive organism focused on either sulfate or metal-reducing conditions.

## Materials and methods

### Biomass preparation

*D. reducens* MI-1 was purchased from the American Type Culture Collection (ATCC® BAA-1160™) and grown anaerobically at 30°C in batch culture with an 80/20 N_2_/CO_2_ headspace in Widdel Low Phosphate (WLP) media (pH 7) as described (Otwell et al., 2015). Cultures were grown with either 28 mM sodium sulfate, 25 mM Fe(III)-citrate, or 35 mM Fe(III)-oxide with 20 mM sodium lactate as electron donor. Fermentation cultures were grown with 20 mM pyruvic acid. Lactate only and Fe(III)-citrate only controls did not exhibit growth. For each cultivation condition analyzed, *D. reducens* was transferred to fresh growth medium at least three times prior to harvesting in order to ensure accurate representation of the proteome for the given cultivation condition. Cell growth was tracked with fluorescence microscopy by staining with acridine orange and the ferrozine assay for quantification of Fe(II) or the Cline assay for quantification of sulfide when appropriate (Lovley and Phillips, 1987; Strocchi et al., 1992). Representative growth curves for each culture condition are displayed in Supplementary Figure 1. Cells were harvested anaerobically using an anaerobic glove bag during mid-late exponential phase and cell pellets were stored at −80°C until protein extraction.

### Protein extraction and digestion

Harvested cell pellets were suspended in 50 mM NH_4_HCO_3_ (pH 8) and lysed in the presence of 100–200 μL of 0.1 mm zirconia/silica beads (BioSpec Products, Bartlesville, OK) as described (Callister et al., 2006) with the following modifications. Bead beating was performed using a Bullet Blender (Next Advance, Averill Park, NY) operated for 3 min at speed 8. The lysate was collected at 4500 g for 5 min at 4°C and the beads were rinsed with 100 μL of 50 mM NH_4_HCO_3_ (pH 8). The combined lysates were transferred to a 1.5-mL microcentrifuge tube and cellular debris was separated from soluble lysate material by centrifuging at 10,000 g for 15 min at 4°C. Protein concentrations within the soluble lysate were measured using the Bicinchoninic Acid (BCA) Protein Assay and then normalized to equal concentration (Smith et al., 1985). Proteins were denatured and digested by following the FASP digest method (Wiśniewski et al., 2009) without alkylation, then washed using C18 SPE chromatography according to previously established protocols (Callister et al., 2008). Equal portions of peptides (100 μg) from biological replicates acquired for each cultivation condition were pooled, then separated using C18 reverse phase high pH fractionation according to previously published protocols (Wang et al., 2011). Ninety-six fractions were collected from each pooled sample and dried overnight in a SpeedVac. Peptides within each well were suspended in 100 ml of 50% methanol and combined into 12 fractions, concentrated to dryness then suspended in nanopure water to achieve a final concentration of 0.1 mg/ml. Additionally, peptides from the unfractionated samples were transferred to MicroSolv ALS vials and diluted with nanopure water to a final concentration of 0.1 μg/μL.

### Proteomic data generation

Mass spectra were generated for both fractionated (60 fractions in total) and unfractionated peptide samples (30 samples originating from 2 to 3 biological replicates per cultivation condition and three technical replicates per biological replicate). The unfractionated samples were randomized and blocked prior to data generation. For both sample types 7 mg of peptides was injected to an Agilent LC system (Agilent Technologies, Santa Clara, CA) coupled to a hybrid ion trap Orbitrap Velos mass spectrometer (Thermo Scientific, San Jose, CA) equipped with an ion funnel and electrospray ionization (ESI) interface. Conditions for peptide separation and HPLC operating conditions have been previously published (Sowell et al., 2008; Robidart et al., 2013). Orbitrap spectra were collected from 400 to 2000 m/z at a resolution of 100 k followed by data-dependent ion trap tandem mass (MS/MS) spectra generation of the six most abundant ions using 35% collision energy (CID). Additional mass spectrometer operating conditions have been previously described (Sowell et al., 2008; Robidart et al., 2013).

### Data analysis

Peptide sequences were assigned to MS/MS spectra using the MSGF search algorithm (Kim et al., 2008) and the translated *D. reducens* MI-1 annotated genome sequence downloaded (September 18, 2012) from the U.S. Department of Energy's Joint Genome Institute's (JGI) IMG database (Markowitz et al., 2014). From measured peptides, an empirical peptide database was generated for use as a library to match high-resolution parent ion spectra (i.e., AMT tag approach Lipton et al., 2002) generated by the Orbitrap Velos instrument from the unfractionated samples. These unfractionated peptide samples were used to obtain label-free abundance measurements for use in relative quantification comparisons. The area under each peptide peak, constructed from ion intensities (ion current) measured across instrument scans, was used to represent the arbitrary abundances of peptides (Lipton et al., 2002; Sowell et al., 2008). The dataset of peptides and their associated abundances, resulting from matching to the library, was filtered to achieve a false discovery rate of =5% using established protocols (Stanley et al., 2011). Peptides matching to multiple proteins were filtered out unless the matched proteins were identical, duplicated genes. Peptide abundances were log transformed (base 2) and protein abundance was estimated as the mean of its measured peptides' log-transformed abundances across replicates. A protein was considered positively observed if identified by at least two unique peptide sequences in replicates from a given cultivation condition. If detected, the protein's mean abundance was compared between different cultivation conditions and *p*-values assigned using ANOVA; part of the InfernoRDN (previously known as DAnTE, Polpitiya et al., 2008) proteomics analysis software tools (http://omics.pnl.gov/software/InfernoRDN). Abundance measurements for a protein were required to be present in at least 50% of replicates in order for its means to be compared. Proteins were considered differentially abundant if their mean peptide intensity had a >2-fold change calculated as the difference between means across the conditions being compared, with a *p* ≤ 0.05. Along with the cultivation conditions described, the AMT tag approach was used to analyze Fe(III)-oxide cultures harvested at 8 days. This data was excluded from the analysis as the 6-day samples matched closer with the growth phase of the other conditions. All raw data is deposited and publicly available in MassIVE (Mass spectrometry Interactive Virtual Environment) with accession number MSV000079501 and ProteomeXchange with accession number PXD003605.

## Results: global proteome of *D. reducens*

The genome of *D. reducens* encodes 3324 predicted proteins, and across the four conditions analyzed in our study, 1268 proteins were confidently identified, representing ~38% of predicted proteins. This includes 1064 proteins with functional annotations and 204 hypothetical proteins. Table 1 displays a global overview of the proteomic data presented.

**Table 1:** **Global overview of *D. reducens* comparative proteomic analysis**.

**Cultivation condition**	**Total proteins identified**	**Average log_2_ peptide ion intensity**	**Proteins unique to condition**	**Proteins significantly increased compared to pyruvate**
Pyruvate fermentation	1104	19.87 (SD 1.49)	113 (Supplementary Table 1a)	NA
Sulfate reduction (lactate as ED)	1046	19.85 (SD 1.63)	81 (Supplementary Table 1b)	109 (Supplementary Table 2a)
Fe(III)-citrate reduction (lactate as ED)	582	19.11 (SD 1.69)	23 (Supplementary Table 1c)	29 (Supplementary Table 2b)
Fe(III)-oxide reduction (lactate as ED)	724	19.58 (SD 1.66)	35 (Supplementary Table 1d)	54 (Supplementary Table 2c)

**Figure 1:**
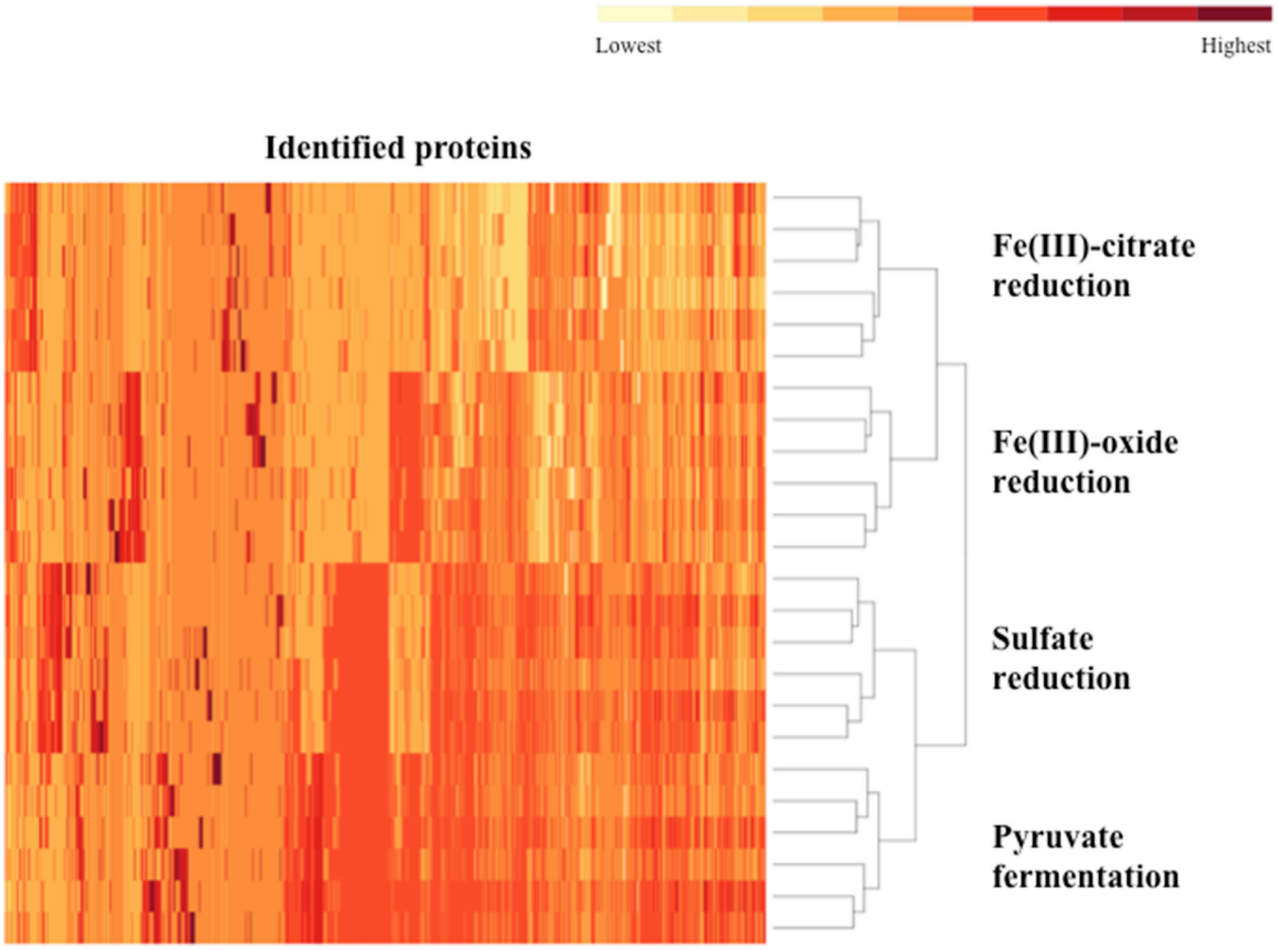
**A heat map of the proteomes generated for *D. reducens***. Replicates grouped together based on hierarchical clustering using the heat map command in R. The two Fe(III) reduction conditions form clusters that are more similar to each other than to any other condition. A similar relationship is observed for the pyruvate fermentation and sulfate reduction condition. Data input for each replicate (two biological duplicates and three technical triplicates per condition) consisted of the average ion intensity for all proteins observed, and data for all detected peptides was included. Identified proteins are displayed across the horizontal axis and ordered by hierarchical clustering with complete linkage. Relative average ion intensity is displayed from yellow (lowest) to red (highest) (R Development Core Team, 2012).

Biological and technical replicates of each condition cluster tightly together when visualizing full-proteome relatedness in a hierarchical clustering-based heat map, demonstrating consistency in the data (Figure 1). A Venn diagram (created in Venny 2.0, Oliveros, 2007) shows overlap of observed proteins across conditions tested, and displays a “core” proteome of 465 proteins identified on every condition (Supplementary Figure 2). In evaluating the number of proteins observed for each cultivation condition, more total proteins were identified on the pyruvate fermentation and sulfate reduction conditions than on the Fe(III) reduction conditions, as there are 1104, 1046, 582, and 724 total proteins observed on pyruvate, sulfate, Fe(III)-citrate, and Fe(III)-oxide respectively (Table 1). 113 proteins were observed solely on the pyruvate condition, 81 on sulfate, 23 on Fe(III)-citrate, and 35 on Fe(III)-oxide (Table 1). These proteins observed exclusively on a single condition are displayed in Supplementary Table 1. Research on model metal-reducing organisms has identified oxidoreductases as well as porin-like proteins that together function as a conduit for extracellular electron transfer, and therefore proteins with either of these predicted functions are highlighted for the Fe(III) reduction conditions (Richardson et al., 2012; Shi et al., 2012; Liu et al., 2014). Proteins that display significant fold increases (>2-fold change, *p* < 0.05) relative to pyruvate during sulfate, Fe(III)-citrate, and Fe(III)-oxide reduction conditions are provided in Supplementary Table 2. This includes 109 proteins significantly increased on sulfate relative to pyruvate, 29 on Fe(III)-citrate, and 54 on Fe(III)-oxide. Again, potential oxidoreductases and porin-like proteins are highlighted for the Fe(III) reduction conditions. A summary of all proteomic data presented in Supplementary Tables is displayed in Table 2.

**Table 2:** **Summary of proteomic data presented in Supplementary Tables**.

**Supplementary Table Number**	**Content**
1a-d	List of proteins unique to each cultivation condition
2a-c	Abundance comparisons for proteins significantly increased compared to pyruvate
3	Abundance comparisons for proteins potentially involved in lactate and/or pyruvate utilization.
4	Abundance comparisons for annotated hydrogenases
5	Abundance comparisons for putative respiration-related proteins
6	Abundance comparisons for additional clusters of proteins significantly increased during sulfate reduction

Along with lower total protein numbers on the Fe(III) reduction conditions, average ion intensities are lower compared to the sulfate and pyruvate conditions. Comparing average log_2_ fold change of average ion intensities across all proteins observed on both pyruvate and sulfate conditions yields a value of ~0.03 (pyruvate to sulfate), demonstrating that neither condition displayed significantly higher ion intensities. However, the average log_2_fold change of all proteins identified on pyruvate and Fe(III)-citrate is 1.34 (pyruvate to Fe(III)-citrate), showing that overall, abundances are >2-fold lower on Fe(III)-citrate. The average log_2_ fold change between the pyruvate and Fe(III)-oxide conditions is 0.78 (pyruvate to Fe(III)-oxide). These differences are reflected in the average ion intensity (log_2_) for all proteins detected, which is 19.87 (*SD* 1.49) for the pyruvate condition, 19.85 (*SD* 1.63) for sulfate, 19.11 (*SD* 1.69) for Fe(III)-citrate, and 19.58 (*SD* 1.66) for Fe(III)-oxide (Table 1). Also reflecting this variance between conditions are abundance patterns of certain housekeeping genes, including the RNA polymerase. For instance, RpoB (Dred_0207) is increased on pyruvate 1.8-fold (*p* < 0.01) relative to sulfate, 5.8-fold (*p* < 0.01) relative to Fe(III)-citrate, and 3.0-fold (*p* < 0.01) relative to Fe(III)-oxide. The β' (Dred_0208) and α (Dred_0243) subunits display similar abundance patterns. The 36 ribosomal proteins encoded in this region are nearly equal in abundance on pyruvate and sulfate (1.1-fold increased on pyruvate relative to sulfate on average), but significantly decreased in abundance on Fe(III) conditions. These ribosomal proteins are increased on average 5.8-fold on pyruvate conditions relative to Fe(III)-citrate and 1.6-fold relative to Fe(III)-oxide. This is likely a reflection of the slower growth rates observed on Fe(III)-reduction conditions compared with sulfate reduction and pyruvate fermentation (Supplementary Figure 1).

**Figure 2:**
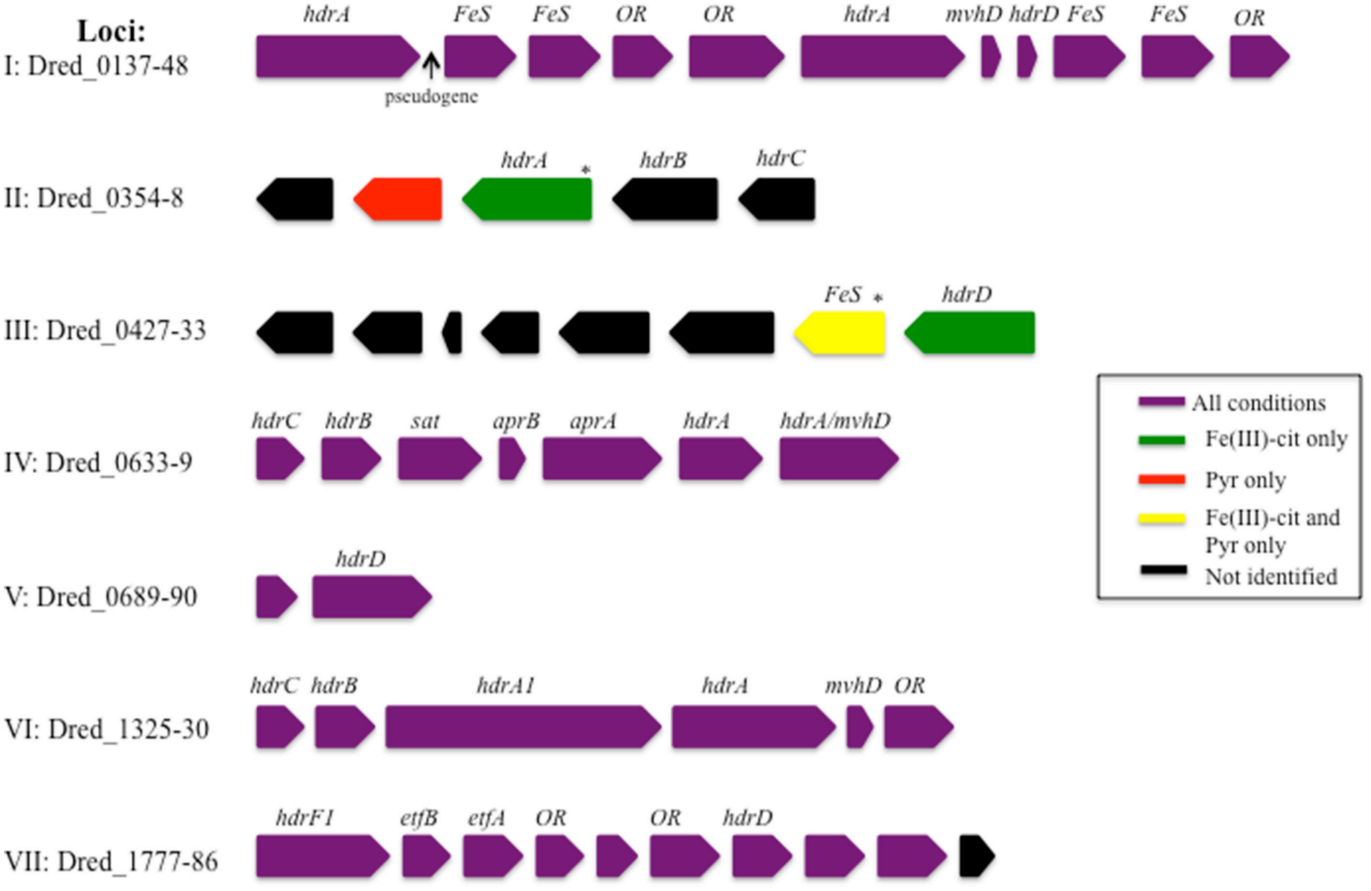
**Identification of proteins encoded within heterodisulfide reductase (*hdr*)-containing loci**. A number of proteins within hdr-containing loci were identified during proteomic analysis of *D. reducens* and many displayed differential abundance across cultivation conditions. Gene name abbreviations stand for iron sulfur proteins (*FeS*), methyl viologen hydrogenase, delta subunit (*mvhD*), annotated oxidoreductases (OR), electron transfer flavoproteins (*etfA* and *etfB*), sulfate adenyltransferase (*sat*), and APS reductase (*aprA* and *aprB*). Subunits of heterodisulfide reductases (*hdr*) are also shown. This figure is modified from an image published in the *D. reducens* genome paper and loci have been renumbered to match with locus tag position in the genome (Junier et al., 2010). ^*^Refers to identification by only one unique peptide in at least half of the replicates.

Our comparative proteomic analysis has revealed many potentially redox-related proteins from *D. reducens*. For instance, seven heterodisulfide reductase (*hdr*)-containing loci were identified in the genome, which are genomic regions rich with other predicted redox-related proteins including ferredoxins, proteins with iron-sulfur binding domains, proteins with flavin-binding domains, and annotated oxidoreductases (Junier et al., 2010). High numbers of unique peptides and differential protein abundance patterns across conditions were observed for proteins encoded within the *hdr*-containing loci. Identification patterns of proteins within these loci are displayed in Figure 2, and abundance comparisons for proteins of interest are displayed in **Table 4**. Proteomic data from various proteins, including proteins within these *hdr*-containing loci, are discussed below.

## Discussion

### Energy production and catabolism of organic carbon in *D. reducens*

**Figure 3:**
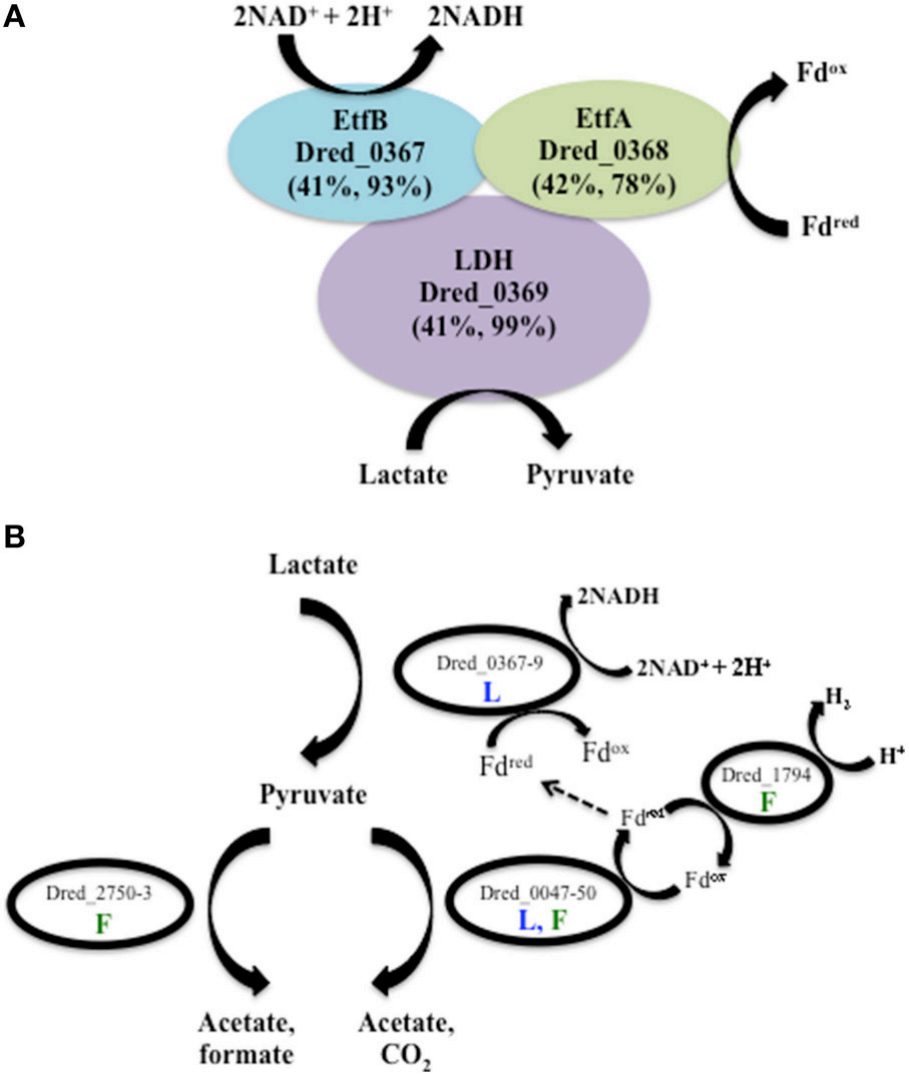
**Predicted pathways of lactate and pyruvate utilization in *D. reducens* based on proteomic analysis. (A)** Dred_0367-9 is proposed to perform lactate oxidation in *D. reducens*. These proteins share similarity with a lactate-oxidizing complex recently described in *Acetobacterium woodii* to operate through flavin-based electron bifurcation (FBEB) (Awo_c08730, 20, and 10; Weghoff et al., 2015). Similarity between *D. reducens* proteins and those from *A. woodii* is displayed as percent sequence identity across percent query coverage (http://blast.ncbi.nlm.nih.gov). **(B)** Model of lactate and/or pyruvate utilization in *D. reducens*. In lactate-fed cultures, lactate is oxidized to pyruvate. Pyruvate is then converted to acetate (along with CO_2_ or formate), and H_2_ is produced during pyruvate fermentation. In lactate-fed conditions, H_2_ is not formed, and instead reduced ferredoxin could be utilized in other pathways, including the lactate oxidation pathway. Pathways predicted to be involved in lactate-fed cultures are denoted with a blue L. Pathways predicted to be involved in pyruvate-fed fermentative cultures are denoted with a green F. Abundance comparisons for proteins displayed are in Supplementary Table 3.

A novel method of lactate metabolism involving a FBEB complex in *Acetobacterium woodii* (Awo_c08730, 20, and 10) was recently described (Weghoff et al., 2015). The researchers suggested that the complex has the same role in many anaerobes, and Dred_0367-9 is the homologous region in *D. reducens*. These proteins are currently annotated as the beta and alpha subunits of electron transfer flavoproteins (Dred_0367-8) and an FAD-linked oxidase domain-containing protein (Dred_0369). A high number of unique peptides for each of these proteins was detected on all conditions, with the lowest number observed on pyruvate. The average ion intensity of each protein in the operon Dred_0367-9 is higher on lactate-fed sulfate reduction cultures than pyruvate fermentation cultures, with *p* < 0.002, 0.08, and 0.03 respectively. Abundance comparisons for all conditions are displayed in Supplementary Table 3. The annotated pathway for lactate oxidation in the *D. reducens* genome contains lactate dehydrogenase (LDH) Dred_2797, but no peptides for this protein were observed in our study. Based on the lack of detection of the annotated LDH, the high sequence similarity between Dred_0367-9 and the FBEB LDH complex, and the confident identification of these proteins in lactate-grown cultures, we propose that Dred_0367-9 is how *D. reducens* oxidizes lactate to pyruvate, producing NADH in the process (Figure 3A). It is important to note that in *A. woodii*, it was proposed that reduced ferredoxin is regenerated from NADH by reverse electron transfer, mediated by the Rnf complex (Weghoff et al., 2015). As the genome of *D. reducens* does not encode this complex, however, reduced ferredoxin would need to be regenerated through other means (see below).

Three potential pathways for pyruvate utilization are predicted in the annotated genome of *D. reducens*, encoded by genes Dred_0047-50, Dred_2750-3, and Dred_1893 (Junier et al., 2010). Proteomic data revealed that the pyruvate-ferredoxin oxidoreductase pathway (Dred_0047-50) is expressed during both lactate oxidation and pyruvate fermentation and that the pyruvate-formate lyase pathway (Dred_2750-3) is expressed only during pyruvate fermentation. The third predicted pathway, through the annotated pyruvate dehydrogenase Dred_1893 (a pseudogene), was not observed (Figure 3B, Supplementary Table 3). The pyruvate-ferredoxin oxidoreductase pathway (Dred_0047-50) yields acetate, CO_2_, and H_2_ during fermentative growth. H_2_ is not produced during lactate oxidation, and instead this pathway is proposed to produce reduced ferredoxin, which could feed into the LDH complex described above (Junier et al., 2010). High numbers of unique peptides were observed for Dred_0047-9 on both lactate and pyruvate-fed cultures and the proteins do not display strong differential abundance patterns across conditions (Supplementary Table 3). Peptides from Dred_0050, an annotated 4Fe-4S ferredoxin, are detected in every condition (and are the highest in abundance on Fe(III)-citrate conditions), but these peptides are also present in another annotated 4Fe-4S ferredoxin (Dred_2822) and therefore cannot be distinguished. The monomeric hydrogenase Dred_1794 was identified as a likely candidate for production of hydrogen during pyruvate fermentation based on transcriptome patterns, and our proteomic data support this hypothesis (Junier et al., 2010). Dred_1794 is significantly increased on pyruvate relative to all other conditions (5 to 8-fold increase, *p* < 0.01). Abundance ratios for this hydrogenase, as well as all six hydrogenases annotated in the genome of *D. reducens*, are displayed in Supplementary Table 4. Our data suggest that the second predicted pyruvate utilization pathway, the acetate and formate yielding pyruvate-formate lyase pathway (Dred_2750-3) is active during pyruvate fermentation. Previously reported experimental evidence supports this finding, as small amounts of formate are shown to accumulate during pyruvate fermentation (Dalla Vecchia et al., 2014b). Dred_2751 is detected only on the pyruvate condition and Dred_2750 and Dred_2752 are most abundant during fermentative growth relative to all other conditions (~3-and 2-fold increased relative to sulfate conditions, *p* < 0.01 and 0.1 respectively). Peptides from Dred_2753 were not detected on any condition (Supplementary Table 3). A model illustrating lactate and/or pyruvate oxidation in *D. reducens* based on proteomic findings is presented in Figure 3B.

The genome of *D. reducens* contains an eleven-subunit, proton-translocating NADH: quinone oxidoreductase (Nuo, Dred_2036-46), predicted to be involved in NADH oxidation for cellular respiration (Junier et al., 2010). On sulfate reduction conditions as well as pyruvate fermentation, four of the subunits were identified (B, C, D, and I), while only the D subunit was observed on Fe(III)-oxide. During Fe(III)-citrate reduction, not a single subunit of Nuo was identified with any confidence, providing evidence that the Nuo complex is not being utilized during this type of growth in *D. reducens*. Cellular respiration generates ATP by ATP synthase, and all but two subunits of the F-type H+-transporting ATPase were identified in our study. The six identified proteins of the ATP synthase (Dred_3149-56) were most abundant during the sulfate reduction condition and decreased in abundance on Fe(III)-citrate relative to all other conditions (Supplementary Table 5). While Fe(III) was initially reported to serve as an electron acceptor for *D. reducens*, more recent reports have suggested that Fe(III) acts as an electron dump rather than a true respiratory electron acceptor (Dalla Vecchia et al., 2014a,b). Our proteomic findings on the Nuo complex as well as the ATP synthase support the idea that *D. reducens* may not be truly respiring Fe(III). We propose that the downregulation of energy production-related proteins during Fe(III) reduction in *D. reducens* indicates that Fe(III) serves as a less suitable cultivation condition for *D. reducens* than sulfate reduction or pyruvate fermentation. This is supported by less vigorous growth of *D. reducens* on Fe(III), as evidenced by substantially lower growth rates and biomass yields (Supplementary Figure 1).

### Proteome of *D. reducens* during sulfate reduction

**Figure 4:**
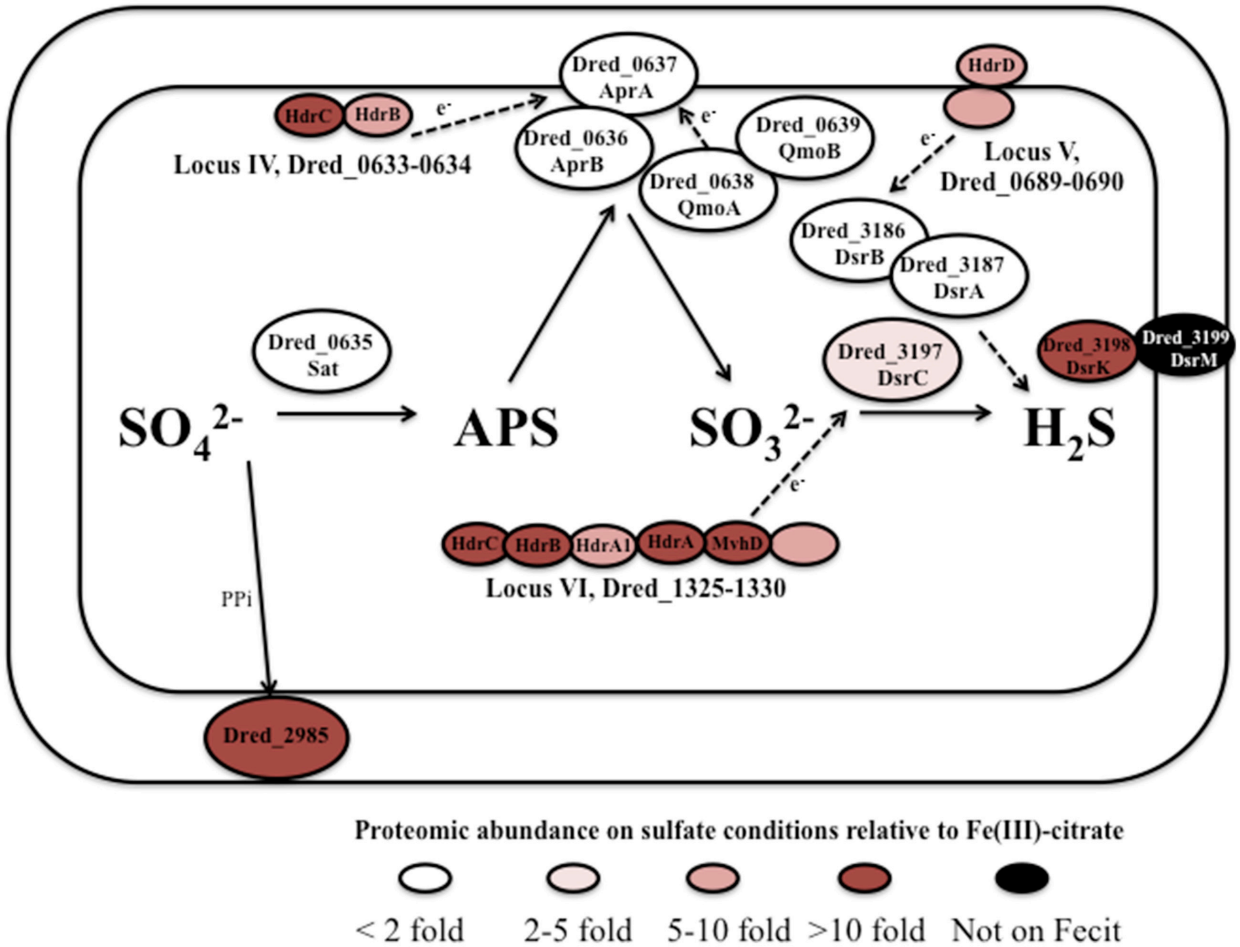
**Predicted pathway of sulfate reduction in *D. reducens* based on proteomic findings**. The core sulfate reduction machinery (Sat, APS reductase, sulfite reductase) does not display significant differential abundance. Instead, certain clusters of proteins encoded within *hdr*-containing loci are increased in abundance during sulfate reduction and are predicted to be involved in the process. Abundance comparisons are shown for sulfate and Fe(III)-citrate reduction conditions. All protein abundance comparisons are displayed in Tables 3, 4.

Our proteomic analysis revealed that core proteins involved in sulfate reduction in *D. reducens* (and conserved between Gram-negative and Gram-positive SROs) are consistent in abundance across conditions. Furthermore, we have identified key clusters of proteins that are highly abundant on sulfate relative to other conditions, and we hypothesize that these are involved in the respiratory process. Some of these clusters include the *hdr*-containing loci introduced previously (Figure 2). Figure 4 summarizes the predicted pathway of electron transfer during sulfate reduction in *D. reducens* based on proteomic evidence.

The core sulfate reduction pathway involves activation of sulfate to adenosine 5′-phosphosulfate (APS) by sulfate adenyltransferase (Dred_0635), processing of the pyrophosphate formed by a pyrophosphatase (Dred_2985), reduction of APS to sulfite by APS reductase (Dred_0636-7), and reduction of sulfite to sulfide by dissimilatory-type sulfite reductase DsrAB (Dred_3186-7, DsrB and DsrA; Pereira et al., 2011; Grein et al., 2013). Our data reveals that in most cases, the core sulfate-reducing machinery is not differentially expressed in *D. reducens* across the cultivation conditions tested (Table 3, Figure 4).

**Table 3:** **Log_2_ abundance comparisons of proteins putatively involved in dissimilatory sulfate reduction in *D. reducens***.

** Locus tag**	**Protein name**	**Log_2_ Sulf/Pyr**	**Log_2_ Sulf/Fe(III)–citrate**	**Log_2_ Sulf/Fe(III)–oxide**	**PSORTb localization**
**CORE SULFATE REDUCTION MACHINERY**					
Dred_0635	Sulfate adenyltransferase (Sat)	−0.28	0.16	−1.03	C
Dred_0636	Adenylylsulfate reductase subunit beta (AprB)	−0.25	0.59	0.27	U
Dred_0637	Adenylylsulfate reductase subunit alpha (AprA)	0.16	0.39	−0.23	C
Dred_2985	Membrane-bound proton-translocating pyrophosphatase	0.40	3.97	1.60	CM
Dred_3186	Sulfite reductase, dissimilatory-type beta subunit (DsrB)	0.42	0.99	0.36	C
Dred_3187	Sulfite reductase, dissimilatory-type alpha subunit (DsrA)	0.26	0.33	0.03	C
**ADDITIONAL PUTATIVE SULFATE REDUCTION PROTEINS**					
Dred_0638	4Fe-4S ferredoxin iron-sulfur binding domain-containing protein (QmoA)	1.07	0.77	0.21	C
Dred_0639	Methyl-viologen-reducing hydrogenase, delta subunit (QmoB)	0.49	0.08	−0.04	C
Dred_3185	Putative dissimilatory sulfite reductase subunit D (DsrD)	−0.02	4.03	3.35	C
Dred_3197	DsrC family protein	0.32	1.43	1.15	C
Dred_3198	Hypothetical protein (DsrK)	−0.90	3.37	0.87	C
Dred_3199	Nitrate reductase, gamma subunit (DsrM)	0.47	NI	−0.14	CM
**Table key:**					
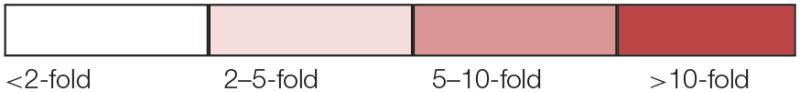					

*Protein identification is based on detection of at least two unique peptides in a biological replicate and peptide detection in at least 50% of replicates. P-values for all ratios highlighted for abundance changes >2-fold are < 0.01. NI, protein not identified. Localization is from PSORTb (Yu et al., 2010). C, cytoplasmic; CM, cytoplasmic membrane; E, extracellular; CW, cell wall; U, unknown*.

Outside of the core enzymes, distinct differences are observed between the genomes of Gram-positive and Gram-negative SROs, as outlined in-depth in recent reviews (Pereira et al., 2011; Grein et al., 2013). In particular, transmembrane complexes that have been ascribed to electron transfer during sulfate reduction in model *Desulfovibrio* species, many of which contain MHC components, are not conserved in Gram-positives. One of these transmembrane complexes is QmoABC, shown to transfer electrons to the APS reductase. The genome of *D. reducens* contains predicted orthologs to QmoA (Dred_0638) and QmoB (Dred_0639), but is missing the transmembrane subunit QmoC, suggesting that other mechanisms are involved in electron transfer to APS reductase. As was observed for the core sulfate-reducing machinery, Dred_0638-9 is similarly abundant across all cultivation conditions (Table 3, Figure 4). Another transmembrane complex of interest is the five-subunit DsrMKJOP, described in *Desulfovibrio* to transfer electrons to the sulfite reductase. The genome of *D. reducens* encodes predicted orthologs only to two subunits and these are rather distant. Dred_3198 shares 37% sequence identity across 81% query coverage to DsrK from *Desulfovibrio vulgaris* and Dred_3199 shares 26% identity across 74% to DsrM (http://blast.ncbi.nlm.nih.gov). Both proteins do not exhibit any discernable differential abundance on sulfate, pyruvate, and Fe(III)-oxide, but Dred_3198 is significantly lower in abundance on Fe(III)-citrate relative to sulfate (10.3-fold higher on sulfate, *p* < 0.01) and Dred_3199 was not identified on Fe(III)-citrate. DsrC (Dred_3197) is a protein conserved in all SROs sequenced to date, positioned next to the Dsr complex, and found to interact with the sulfite reductase (Venceslau et al., 2014). DsrC was recently shown to form a trisulfide intermediate and to serve as a co-substrate for DsrAB (Santos et al., 2015). In *D. reducens*, DsrC is increased >2-fold on sulfate vs. both Fe(III) conditions (Table 3).

#### Proteins in heterodisulfide reductase-containing loci are abundant in sulfate reduction proteome

Our proteomic analysis revealed specific clusters of proteins that are abundant during sulfate reduction relative to the other cultivation conditions. Interestingly, three of these clusters fall into the *hdr*-containing loci displayed in Figure 2, specifically Dred_0633-4, Dred_0689-90, and Dred_1325-30 (Table 4). Dred_0633-4, a predicted operon in locus IV, is significantly increased relative to Fe(III) reduction conditions but not pyruvate fermentation conditions. However, the positioning of Dred_0633 (an HdrC-type protein) and Dred_0634 (an HdrB-type protein) next to key sulfate reduction proteins including the sulfate adenyltransferase (Dred_0635), the APS reductase subunits (Dred_0636-7, AprBA), and QmoAB (Dred_0638-9) suggests an involvement in sulfate reduction. Due to their genomic localization near the AprBA, these Hdr-like proteins may be involved in electron transfer to this reductase (Figure 4). In fact, in all sequenced *Desulfotomaculum* genomes, orthologs of these two Hdr-like proteins exist next to the sulfate adenyltransferase and APS reductase, as determined by the IMG neighborhood viewer (https://img.jgi.doe.gov).

**Table 4:** **Log_2_ proteomic abundance comparisons for proteins discussed from heterodisulfide reductase (*hdr*)-containing loci**.

**Locus tag**	**Protein name**	**Log_2_ Sulf/Pyr**	**Log_2_ Sulf/Fecit**	**Log_2_ Sulf/Feox**	**Localization**
**LOCUS IV: Dred_0633-4, SULFATE-INDUCED**					
Dred_0633	Putative heterodisulfide reductase, C subunit (HdrC)	−0.33	2.57 (<0.01)	2.62	C
Dred_0634	CoB–CoM heterodisulfide reductase (HdrB)	0.23	2.27 (<0.01)	1.02	C
**LOCUS V: Dred_0689-90, SULFATE-INDUCED**					
Dred_0689	Hypothetical protein	0.23	3.76 (<0.01)	2.74	C
Dred_0690	hypothetical protein (HdrD)	0.01	2.35 (<0.01)	1.05	CM
**LOCUS VI: Dred_1325-30, SULFATE-INDUCED**					
Dred_1325	Heterodisulfide reductase, C subunit (HdrC)	1.88 (<0.01)	6.02 (<0.01)	0.06	C
Dred_1326	Hypothetical protein (hdrB)	1.25 (<0.01)	4.1 (<0.01)	3.81 (<0.01)	C
Dred_1327	4Fe-4S ferredoxin iron-sulfur binding domain-containing protein (HdrA1)	1.03 (<0.01)	2.98 (<0.01)	2.54 (<0.01)	C
Dred_1328	4Fe-4S ferredoxin iron-sulfur binding domain-containing protein (HdrA)	0.67 (<0.01)	4.36 (<0.01)	2.9 (<0.01)	C
Dred_1329	Methyl-viologen-reducing hydrogenase, delta subunit (MvhD)	0.75 (<0.01)	4.6 (<0.01)	4.1 (<0.01)	U
Dred_1330	Formate dehydrogenase	0.85 (<0.01)	3.09 (<0.01)	2.11 (<0.01)	C
**Locus tag**	**Protein name**	**Log_2_ Fecit/pyr**	**Log_2_ Fecit/Sulf**	**Log_2_ Fecit/Feox**	**Localization**
**LOCUS I: NO SIGNIFICANT DIFFERENTIAL ABUNDANCE PATTERN**					
Dred_0137/0143	4Fe-4S ferredoxin iron-sulfur binding domain-containing protein (HdrA)	−0.29	−0.77 (<0.01)	−0.24	U
Dred_0138	pseudogene	NI	NI	NI	
Dred_0139/0146	4Fe-4S ferredoxin iron-sulfur binding domain-containing protein (FeS)	−0.85 (0.01)	−0.62	0.71 (0.04)	CM
Dred_0140/0147	4Fe-4S ferredoxin iron-sulfur binding domain-containing protein (FeS)	−0.26	−0.60 (0.01)	−0.47 (0.02)	C
Dred_0141/0148	Oxidoreductase FAD/NAD(P)-binding subunit	−1.07 (<0.01)	−1.61 (<0.01)	0.09	C
Dred_0142	Hypothetical protein	−0.96 (<0.01)	−1.28 (<0.01)	1.48 (<0.01)	C
Dred_0144	Methyl-viologen-reducing hydrogenase, delta subunit (MvhD)	1.37	0.62	0.47	U
Dred_0145	Heterodisulfide reductase subunit (HdrD)	−0.7 (0.03)	−1.61 (<0.01)	−0.43	U
**LOCUS III: Dred_0432-3, Fe(III)-CITRATE-INDUCED**					
Dred_0432	Hypothetical protein (FeS)	Fecit only (1 unique pep)			C
Dred_0433	CoB–CoM heterodisulfide reductase (HdrD)	1.80 (<0.01)	NI	NI	CM
**LOCUS VI: Dred_1778-84, Fe(III)-CITRATE-INDUCED**					
Dred_1778	Electron transfer flavoprotein subunit beta (EtfB)	0.01	−0.02	2.73 (<0.01)	C
Dred_1779	Electron transfer flavoprotein subunit alpha (eEfA)	0.23	0.24	3.58 (<0.01)	U
Dred_1780	3-hydroxybutyryl-CoA dehydrogenase	1.22 (<0.01)	0.39 (0.01)	3.29 (<0.01)	C
Dred_1781	Enoyl-CoA hydratase/isomerase	2.12 (<0.01)	2.12 (<0.01)	4.07 (<0.01)	C
Dred_1782	butyryl-CoA dehydrogenase	0.62 (0.02)	−0.17	1.84 (<0.01)	C
Dred_1783	Hypothetical protein (HdrD)	0.98 (<0.01)	0.87 (<0.01)	1.87 (<0.01)	CM
Dred_1784	Acetyl-CoA acetyltransferase	0.71 (<0.01)	0.41	3.49 (<0.01)	C

*Protein identification is based on detection of at least two unique peptides in a biological replicate (unless noted otherwise) and peptide detection in at least 50% of replicates. All significant p-values (<0.05) are shown. NI, protein not identified. Localization is from PSORTb (Yu et al., 2010). C, cytoplasmic; CM, cytoplasmic membrane; E, extracellular; CW, cell wall; U, unknown*.

A significant increase in abundance is also observed for the predicted operon Dred_0689-90 on the sulfate reduction condition relative to Fe(III) reduction conditions but not relative to pyruvate conditions. Dred_0689 is annotated as a hypothetical protein and Dred_0690 is an HdrD-like protein (Figure 2 and Table 4). Both of these proteins have annotations that suggest involvement in lactate oxidation, described by IMG as L-lactate utilization proteins LutC and LutB respectively, which suggests an involvement in lactate-fed sulfate reduction rather than pyruvate fermentation. Furthermore, the *hdr*-containing locus is conserved across *Desulfotomaculum* species, and several species contain an adjacent L-lactate transport protein. The locus is also conserved in species of *Desulfosporosinus* and *Desulfitobacterium* (the latter of which reduces sulfite but not sulfate), suggesting that these proteins may be involved with lactate utilization and/or electron transfer to the sulfite reductase (https://img.jgi.doe.gov).

**Figure 5:**
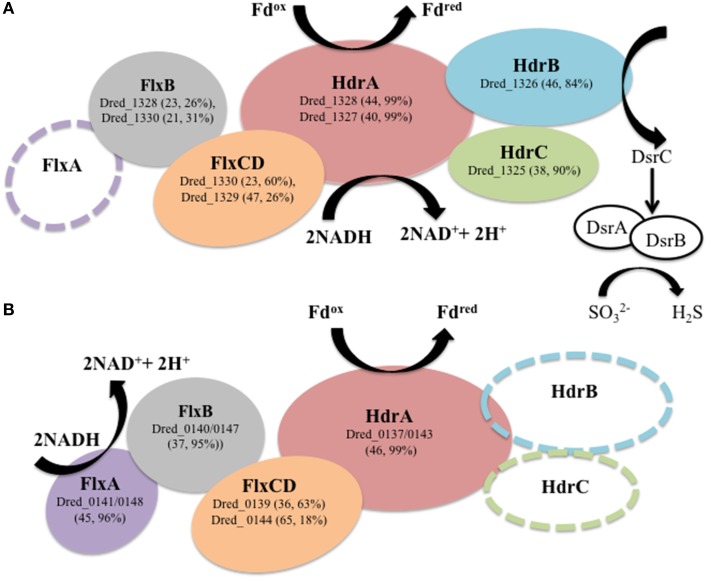
***Hdr*-containing locus VI (Dred_1325-30) is proposed to transfer electrons to DsrC. (A)** Dred_1325-30 is significantly increased in abundance during sulfate reduction relative to other cultivation conditions analyzed. This locus contains orthologs to proteins within the flavin-based electron bifurcation (FBEB) FlxABCD-HdrABC cluster described in *Desulfovibrio vulgaris* Hildenborough (DVU2399-2404) to be involved in electron transfer to DsrC (Ramos et al., 2015). Sequence similarity is displayed as percent sequence identity across percent query coverage (http://blast.ncbi.nlm.nih.gov). An open circle displays lack of an ortholog. (**B**) *Hdr*-containing locus I (Dred_0137-148) contains similarity to several proteins within locus VI and the FlxABCD-HdrABC cluster but is missing two of the Hdr-type proteins. Dred_0141 and Dred_0148 (identical proteins) are orthologs to the FlxA protein missing from the Dred_1325-30 cluster. It is possible that locus VI (Dred_1325-30) and locus I (Dred_0137-48), both of which were identified with high numbers of unique peptides in our proteomic data, work together to carry out FBEB in order to reduce DsrC.

A particularly striking *hdr*-containing locus is Dred_1325-30 (locus VI in Figure 2), where a significant increase in abundance is observed across the six proteins on sulfate relative to all other cultivation conditions (with *p* < 0.05; Table 4). For instance, compared with the Fe(III)-citrate condition, these proteins are increased >20-fold on average. The six proteins of this locus are completely conserved only in other species of *Desulfotomaculum* that are incomplete lactate oxidizers (https://img.jgi.doe.gov). Intriguingly, there is similarity between Dred_1325-30 and the FlxABCD-HdrABC cluster, recently shown in *Desulfovibrio vulgaris* Hildenborough to be essential for NADH oxidation during sulfate reduction with ethanol as electron donor and suggested to reduce DsrC through FBEB (Ramos et al., 2015). The Hdr components are completely conserved, as are parts of the Flx components. While Dred_1325-30 is missing an ortholog to FlxA (the predicted NADH dehydrogenase), Dred_1327 is a much larger protein than the HdrA from *Desulfovibrio* and contains multiple partial HdrA domains as well as putative NADH-binding domains (MicrobesOnline, Dehal et al., 2010). This large protein may be performing the NADH-oxidizing function in the cluster, while Dred_1328 (also an HdrA-type protein) reduces ferredoxin (Figure 5A). Therefore, it is possible that this cluster may be performing a similar function in *D. reducens*, utilizing FBEB in order to improve the energetic favorability of sulfate reduction. Another *hdr*-containing locus in *D. reducens* (Dred_0137-48) shows similarity to the Dred_1325-30 cluster and in fact encodes components of Flx missing from Dred_1325-30 (Figure 5B). Dred_0137-48 is missing the HdrB and HdrC components of the cluster, but it is possible that this locus coordinates with Dred_1325-30, oxidizing NADH and working together to reduce ferredoxin and DsrC. The Dred_0137-48 locus is not differentially abundant across conditions in most cases (see further description below), unlike the Dred_1325-30 cluster (Table 4). Additional clusters of interest on the sulfate reduction condition (not *hdr*- containing) are displayed in Supplementary Table 6.

### Proteome of *D. reducens* during Fe(III) reduction

All previous RNA and/or protein-based analyses of Fe(III) reduction in *D. reducens* have used soluble Fe(III) due to technical challenges associated with culturing and sample preparation on insoluble Fe(III) (Dalla Vecchia et al., 2014a,b). In this study, we have analyzed the proteomes of *D. reducens* cultivated on both soluble Fe(III)-citrate and insoluble Fe(III)-oxide, allowing for a valuable comparison of external electron acceptors.

#### Proteins in heterodisulfide reductase-containing loci are abundant in soluble Fe(III) reduction proteome

**Figure 6:**
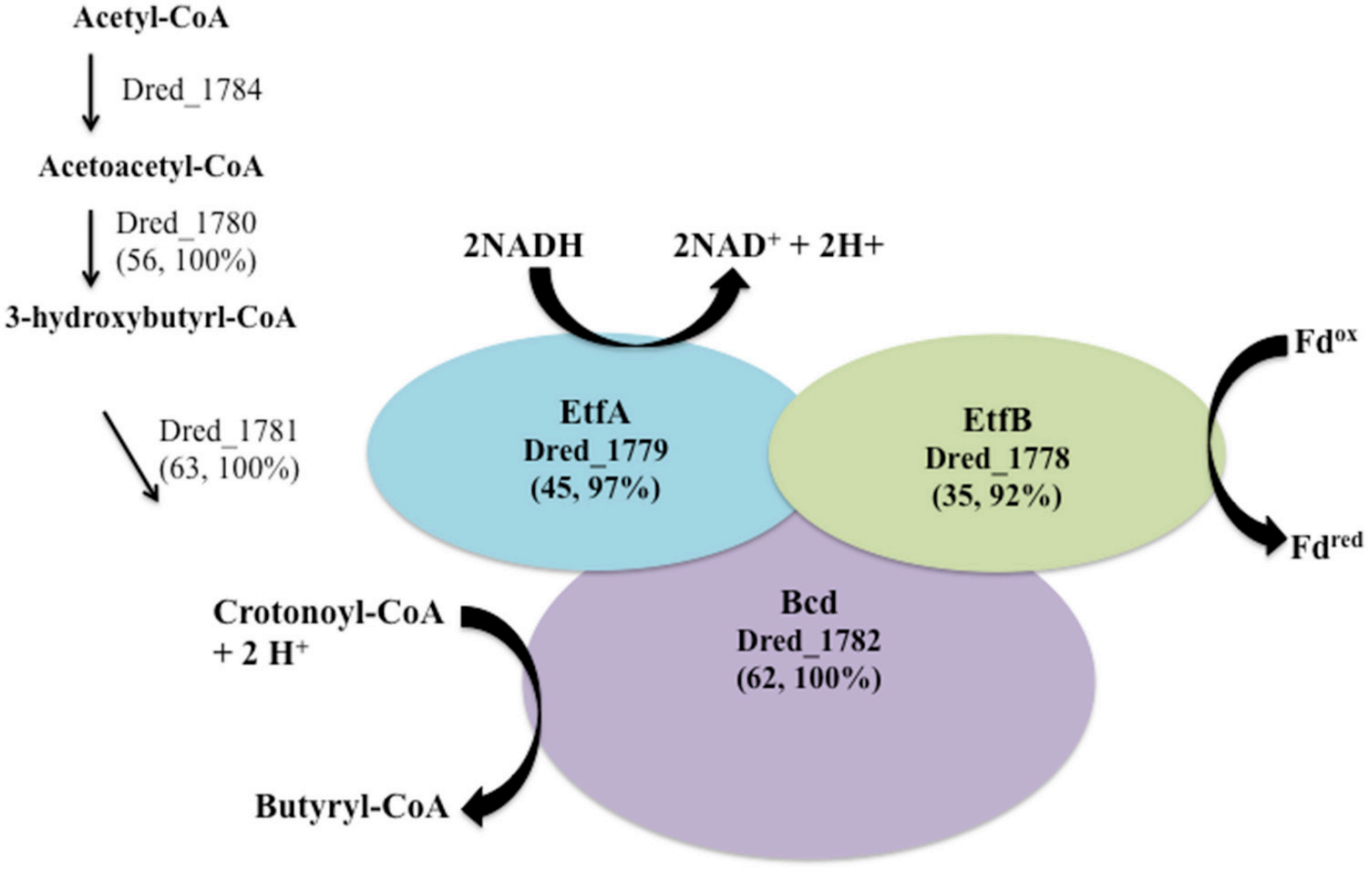
**The cluster of proteins most increased on Fe(III)-citrate relative to pyruvate fermentation has similarity to a described FBEB system**. A significant number of peptides and overall increased abundance was observed for proteins within *hdr*-containing locus VII (Dred_1778-84) on the Fe(III)-citrate condition. Genes within this locus are orthologs to the FBEB complex BcdA–EtfBC from *Clostridium kluyveri* DSM 555 (Li et al., 2008; Buckel and Thauer, 2013). Similarity between this complex (CKL_0454-8) and proteins in *D. reducens* is displayed as percent sequence identity across percent query coverage (http://blast.ncbi.nlm.nih.gov). Furthermore, redundant genes for each of the proteins involved in the FBEB step are expressed solely on the Fe(III)-citrate condition and include the electron transfer flavoproteins Dred_0573 and Dred_0572 (45, 97% and 31, 80%), and the acyl-CoA dehydrogenase domain-containing protein Dred_0402 (53, 100%). Furthermore, the enoyl-CoA hydratase/isomerase Dred_0401 is a redundant protein (60, 100%) for the step leading to crotonoyl-CoA, also unique to the Fe(III)-citrate condition.

Three *hdr*-containing loci are highlighted in the proteome of *D. reducens* during soluble Fe(III) reduction. This includes proteins within locus VII (Dred_1777-86) locus III (Dred_0427-33), and locus I (Dred_0137-48) (Figure 2). While the electron transfer proteins (Dred_1778-9) are similarly abundant on Fe(III)-citrate, sulfate, and pyruvate, Dred_1780-4 represents the most increased cluster of three or more proteins on Fe(III)-citrate relative to pyruvate (Table 4). It is increased in abundance ~2.2-fold across the five proteins (all *p* < 0.02). In addition, while the Fe(III)-citrate condition overall has nearly half of the total unique peptides observed on the sulfate and pyruvate condition, and accordingly tends to have less peptides detected for each protein, the number of unique peptides detected across Dred_1778-84 is significantly higher on Fe(III)-citrate than all other conditions. Averaged across technical triplicates and biological duplicates, the mean number of peptides/proteins detected across the seven proteins is 42.9 on the Fe(III)-citrate condition, 17.2 on pyruvate, 20.8 on sulfate, and 8.0 on Fe(III)-oxide. Five proteins in this cluster (Dred_1782, Dred_1784, and Dred_1778-80) fall into the top 20 proteins across the Fe(III)-citrate proteome with respect to highest unique peptide counts. This result is surprising because these genes are predicted to be involved in butyrate oxidation, which is not the electron donor for any cultures analyzed in this study. It is possible that these proteins are using a different substrate than their annotation suggests, or that these enzymes are acting in the opposite direction, converting acetyl-CoA to butyrl-CoA (Figure 6). In fact, proteins within this cluster are similar to one of the most well-described FBEB systems, the clostridial butyryl-CoA dehydrogenase/electron transferring flavoprotein (BcdA–EtfBC) complex (Li et al., 2008; Buckel and Thauer, 2013). This complex is known to catalyze the electron bifurcation from NADH to ferredoxin and crotonyl-CoA. The proteins Dred_1778-9 (electron transfer flavoproteins) and Dred_1782 (butyryl-CoA dehydrogenase) match closely to this complex, while orthologs to Dred_1780-2 and Dred_1784 catalyze the reactions leading to this FBEB. In this scheme, a protein such as the formate acetyltransferase Dred_0039 (observed on all conditions and most abundant on pyruvate) could be converting pyruvate to acetyl-CoA. Then, following FBEB, reduced ferredoxin would be available for other reductive processes in the cell. A full visual comparing the BcdA-EtfBC complex in *Clostridium kluyveri* DSM 555 with the *D. reducens* locus is displayed in Figure 6 (Buckel and Thauer, 2013). *D. reducens* also contains proteins that are redundant to components of this FBEB system and these are unique to the Fe(III)-citrate condition. Also important to note, the described clostridial FBEB system lacks Hdr-like proteins. The placement of the HdrD-like protein Dred_1783 in this annotated butyrate-oxidizing cluster is unique to *D. reducens* (https://img.jgi.doe.gov).

In the *hdr*-containing locus III (Dred_0427-33), peptides for proteins encoded between Dred_0427-31 were not identified in our study. Dred_0432-3, however, is significant on the Fe(III)-citrate condition (Table 4). Dred_0432 was only identified on pyruvate and Fe(III)-citrate and is increased ~3.5-fold on Fe(III)-citrate (*p* < 0.01). Dred_0433 is completely unique to the Fe(III)-citrate condition. The identification of Dred_0432 on Fe(III)-citrate is based on a single unique peptide in 5/6 replicates. Dred_0433 is localized to the cytoplasmic membrane as predicted by PSORTb (Yu et al., 2010). The predicted operon is from Dred_0431-3, and Dred_0431 is a predicted permease. Dred_0432-3 encompasses the Hdr component of the locus, and interestingly, both proteins have similarity to the L-lactate utilization protein LutB, as described by IMG. As additional evidence toward a role of the proteins in Fe(III) reduction, the closest orthologs to Dred_0432-3 are all from other Gram-positive metal-reducers, specifically *Desulfosporosinus* and *Desulfitobacterium* species. In these other genera, the genes are encoded next to five permease/transport proteins (https://img.jgi.doe.gov). Dred_0432-3 is not conserved in other *Desulfotomaculum* species.

The *hdr*-containing locus Dred_0137-48 is also of interest. While many proteins within the locus are similar to Dred_1325-30 (Figure 5) our proteomic abundance data reveals that Dred_0137-48 is not strongly differentially abundant across conditions and that a high number of unique peptides are observed on all conditions (Table 4). Interestingly, the Dred_0137-148 locus contains one of three redox proteins predicted as putative Fe(III) reductases in the analysis of the surfaceome of *D. reducens* (Dalla Vecchia et al., 2014a). This is the ferredoxin Dred_0143, which has an unknown localization based on PSORTb, but contains one transmembrane helix as predicted by the transmembrane helix (TMH) prediction algorithm TMHMM Server v. 2.0 (www.cbs.dtu.dk/services/TMHMM). A duplication is encoded within this locus which is only observed in two other *Desulfotomaculum* species, and Dred_0137 is identical to Dred_0143. The authors did not observe significant differential abundance of this protein between conditions tested (pyruvate fermentation and Fe(III)-citrate with lactate as electron donor), which is consistent with our findings (Table 4). The high expression of this locus on all conditions (in contrast to the downregulation of Dred_1325-30 on Fe(III) conditions) supports the possible involvement of Dred_0143 in Fe(III) reduction proposed by the surfaceome study (Dalla Vecchia et al., 2014a). The other two proteins identified in the surfaceome study as potential Fe(III) reductases are not supported by our proteomic data. This includes Dred_0462, a subunit of the membrane-bound, trimeric hydrogenase Dred_0461-3 (Supplementary Table 3). Dred_0462 was suggested as a putative Fe(III) reductase, although the authors observed a decrease in its abundance during Fe(III)-reduction relative to pyruvate fermentation. Similarly in our study, both Dred_0462 and Dred_0463 (the catalytic domain) are increased during pyruvate fermentation relative to Fe(III)-reduction and most abundant during sulfate-reduction (with *p*-values of 0.02 and 0.01 relative to pyruvate; Supplementary Table 3). From these findings, it is most likely that this hydrogenase is utilized during sulfate reduction and/or pyruvate fermentation by *D. reducens*. Finally, the only redox-related protein that increased in abundance during Fe(III)-citrate reduction vs. pyruvate fermentation in the surfaceome study was the alkyl hydroperoxide reductase Dred_1533. We observed no peptides for this protein on any condition.

#### Additional proteins of interest on the Fe(III) reduction condition

Dred_0701, annotated as the sole multiheme c-type cytochrome (Dred_0700-1) was identified only on the Fe(III)-oxide condition. A single peptide was identified in four out of six replicates, whereas no peptides were detected from this protein on any other condition. This is the first evidence of metal reduction-related MHC expression in *D. reducens*. This finding emphasizes the importance of testing environmentally relevant conditions like insoluble Fe(III), as previous analyses of the MHC were performed during soluble Fe(III) and U(VI) reduction. Potentially related to this finding, the protein most increased in abundance on Fe(III)-oxide relative to pyruvate is porphobilinogen deaminase (Dred_2163, HemC), a protein in the heme biosynthesis pathway (~136.1-fold increase, *p* < 0.01). This protein was identified by a single peptide in each replicate of the Fe(III)-oxide condition. Two other proteins in this pathway are also highest in abundance on the Fe(III)-oxide condition relative to all other conditions (Dred_2160 and Dred_2162, HemB and HemD respectively).

Finally, our comparative proteomic analysis provides support for the involvement of the NADH:flavin oxidoreductase (Dred_2421) in Fe(III) reduction, a protein that our group previously identified as a metal reductase based on functional screens of the *D. reducens* proteome (Otwell et al., 2015). Multiple unique peptides were observed for this protein only on Fe(III)-oxide, so therefore with stringent filtering criteria Dred_2421 is unique to the Fe(III)-oxide condition (Supplementary Table 1d). Including data for proteins identified by a single unique peptide in at least 50% of the replicates, Dred_2421 is actually most abundant on the Fe(III)-citrate condition, increased ~1.6-fold (*p* < 0.01) relative to pyruvate and ~2.3-fold (*p* < 0.01) relative to the sulfate condition. Comparing the single peptide identified on all conditions, Dred_2421 is ~3 fold more abundant on Fe(III)-citrate than Fe(III)-oxide. The closest orthologs to this protein are not from *Desulfotomaculum* species, but instead from other genera of Gram-positive metal reducers, specifically *Desulfitobacterium* and *Deuslfosporosinus* species. Interestingly, in *Desulfitobacterium* species, this NADH:flavin oxidoreductase is inserted within the *hdr*-containing locus orthologous to Dred_1778-84 (https://img.jgi.doe.gov).

## Conclusions

Comparative proteomic analysis of *D. reducens* cultivated on varied conditions has revealed multiple insights into the metabolism of this Gram-positive organism. Our proteomic dataset allows us to analyze predictions made by the annotated genome and form stronger hypotheses about protein function. A greater number of proteins were observed while *D. reducens* was either reducing sulfate (with lactate as electron donor) or fermenting pyruvate compared with Fe(III)-reducing conditions. The soluble and insoluble Fe(III)-reducing proteomes, analyzed for the first time in a Gram-positive organism, were distinctive from one another, a result consistent with findings in the Gram-negative metal-reducing organisms *Geobacter sulfurreducens* and *Geobacter bemidjiensis* (Ding et al., 2008; Merkley et al., 2015). Peptides for the sole MHC annotated in the genome (Dred_0700-1) were detected only on the insoluble Fe(III) condition, and an enzyme involved in heme biosynthesis was upregulated >100-fold on Fe(III)-oxide relative to pyruvate. Certain clusters of proteins were significantly differentially abundant across cultivation conditions studied. Several of these clusters include Hdrs, and our study has suggested potential involvement of these *hdr*-containing loci in metabolic processes in *D. reducens* including sulfate and Fe(III) reduction.

While comparative expression analyses (mRNA and protein-based) are accepted methods for highlighting genes/proteins of interest, these approaches have limitations. Namely, differential expression is not necessarily directly linked with function (Price et al., 2013). For instance, our analysis of putative sulfate reduction-related proteins in *D. reducens* revealed that most of these proteins are not differentially abundant on cultivation conditions tested, which is not the result we expected. Nonetheless, comparative proteomic analysis offers a method for hypothesis generation regarding protein function and is especially useful in poorly characterized organisms such as *D. reducens*.

## Author contributions

AO participated in conception of the study, experimental work, analysis and interpretation of proteomic data, and wrote the first draft of the manuscript. SC participated in conception of the study, global proteomic analysis, and reviewed and edited the manuscript. EZ participated in global proteomic analysis and reviewed and edited the manuscript. RS participated in global proteomic analysis and reviewed and edited the manuscript. RR participated in conception of the study, global proteomic analysis, and reviewed and edited the manuscript.

## Funding

This project was funded by the Department of Energy's Office of Biological and Environmental Research within the Office of Science, project number DE-SC0006644. A portion of this research was funded by the Department of Energy's (DOE) Office of Biological and Environmental Research (OBER) Pan-omics program and performed in the Environmental Molecular Sciences Laboratory at Pacific Northwest National Laboratory (PNNL). The Environmental Molecular Sciences Laboratory is a U.S. Department of Energy (DOE) Office of Biological and Environmental Research national scientific user facility on the PNNL campus. PNNL is a multi-program national laboratory operated by Battelle for the DOE under contract DE-AC05-76RL01830.

### Conflict of interest statement

The authors declare that the research was conducted in the absence of any commercial or financial relationships that could be construed as a potential conflict of interest.
