# Supplementary figures and images for: Comparative Proteomic Analysis of Desulfotomaculum reducens MI-1: Insights into the Metabolic Versatility of a Gram-Positive Sulfate- and Metal-Reducing Bacterium
Source: Front Microbiol. 2016 Feb 19;7:191. doi: 10.3389/fmicb.2016.00191 (PMC4759654; doi:10.3389/fmicb.2016.00191)

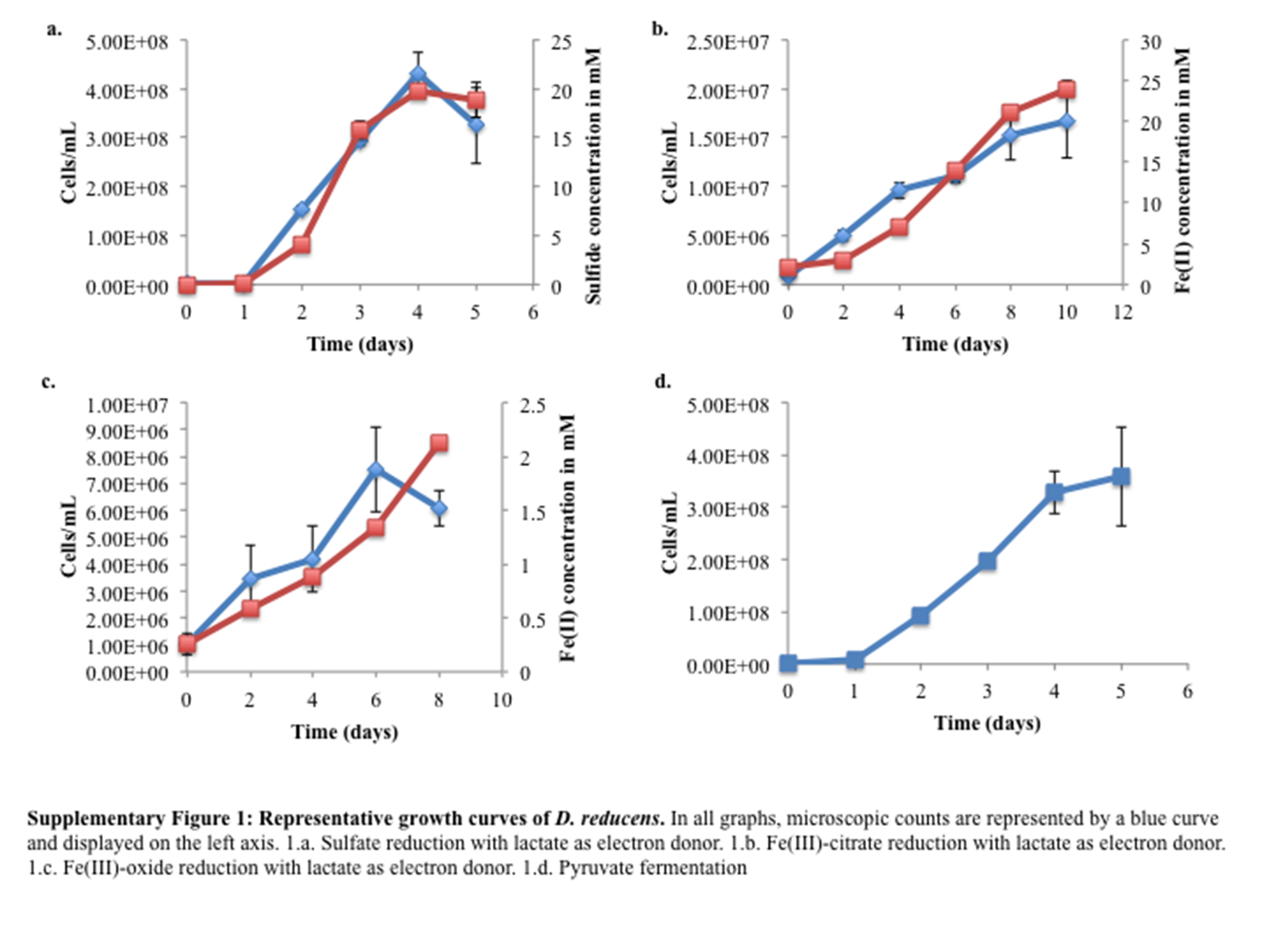

Supplement: Supplementary file 2 [file Image1.TIFF]

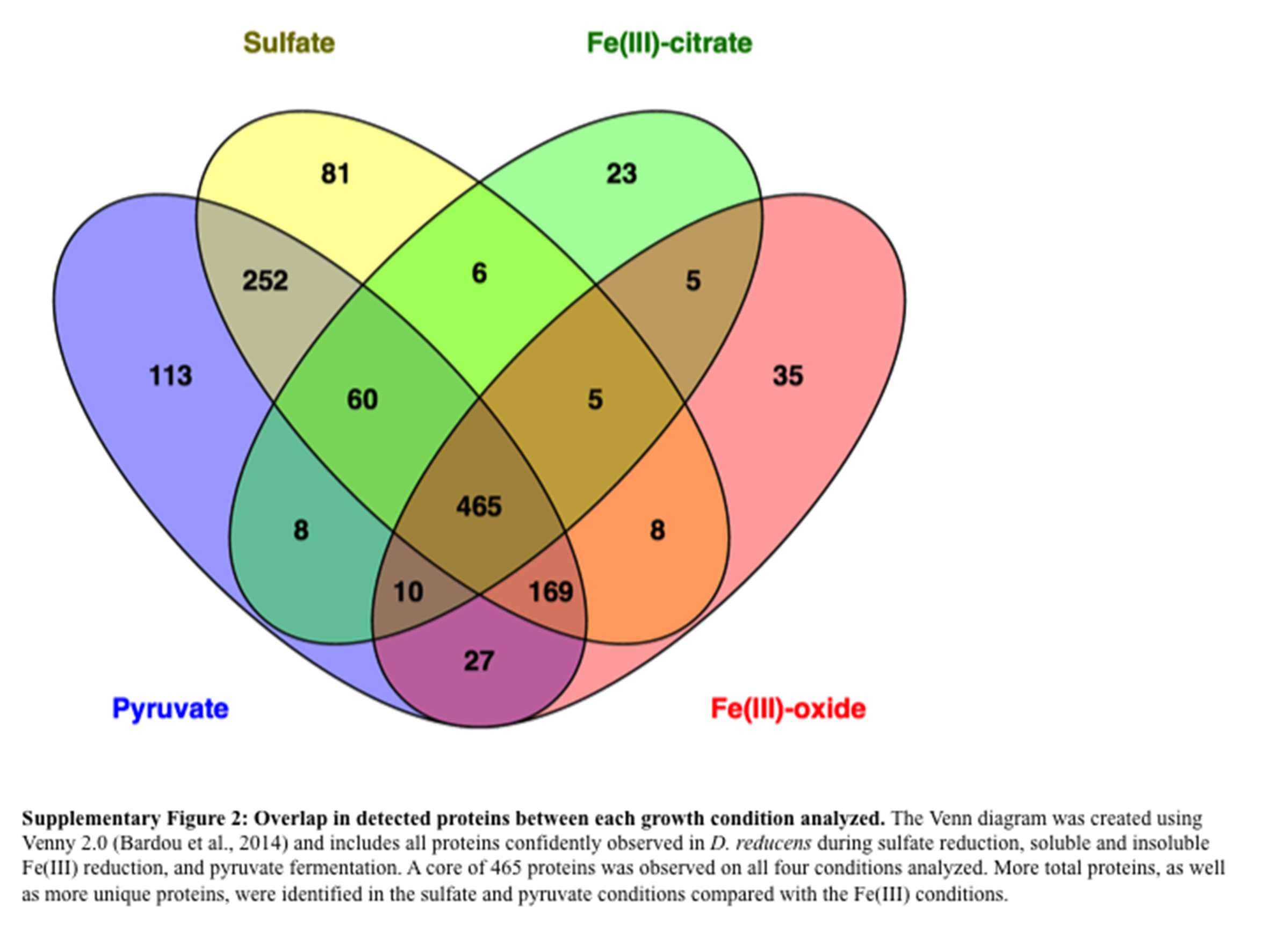

Supplement: Supplementary file 3 [file Image2.TIFF]
